# NAD(P)-dependent steroid dehydrogenase-like is involved in breast cancer cell growth and metastasis

**DOI:** 10.1186/s12885-020-06840-2

**Published:** 2020-05-04

**Authors:** So-Hyun Yoon, Hoe Suk Kim, Ryong Nam Kim, So-Youn Jung, Bok Sil Hong, Eun Ji Kang, Han-Byoel Lee, Hyeong-Gon Moon, Dong-Young Noh, Wonshik Han

**Affiliations:** 1 Seoul National University College of Medicine, 101 Daehak-ro, Jongno-gu, Seoul, 03080 Republic of Korea; 2 Biomedical Research Institute, Seoul National University Hospital, 101 Daehak-ro, Jongno-gu, Seoul, 03080 Republic of Korea; 3 Department of Surgery, Seoul National University Hospital, 101 Daehak-ro, Jongno-gu, Seoul, 03080 Republic of Korea; 4 Center for Breast Cancer, National Cancer Center, 323 Ilsan-ro, Ilsandong-gu, Goyang-si, Gyeonggi-do 10408 Republic of Korea; 5 Cancer Research Institute, Seoul National University, 101 Daehak-ro, Jongno-gu, Seoul, 03080 Republic of Korea

**Keywords:** Breast cancer, NSDHL, Knockdown, Proliferation, Metastasis, Cholesterol, EGFR

## Abstract

**Background:**

The cholesterol biosynthesis pathway is typically upregulated in breast cancer. The role of NAD(P)-dependent steroid dehydrogenase-like (NSDHL) gene, which is involved in cholesterol biosynthesis, in breast cancer remains unknown. This study aimed to uncover the role of NSDHL in the growth and metastasis of breast cancer.

**Methods:**

After NSDHL knockdown by transfection of short interfering RNA into human breast cancer cell lines (MCF-7, MDA-MB-231 and BT-20) and human breast epithelial cell line (MCF10A), cell proliferation assay, cell cycle analysis, three-dimensional cell culture, clonogenic assay, transwell migration and invasion assays, and wound healing assay were performed. Erlotinib was used as the target drug for epidermal growth factor receptor. Immunodeficient mice (NOD.Cg-Prkdcscid Il2rgtm1wjl /SzJ) were used as orthotropic breast tumor models by injecting them with NSDHL-knockdown MDA-MB-231 cells using lentivirus-carrying NSDHL short hairpin RNA. Clinical data from 3951 breast cancer patients in Gene Expression Omnibus databases were used to investigate the potential prognostic role of NSDHL by survival analysis.

**Results:**

NSDHL knockdown in BT-20, and MDA-MB-231 resulted in a significant decrease in their viability, colony formation, migration, and invasion abilities (*p* < 0.05). Total cholesterol levels were observed to be significantly decreased in NSDHL-knockdown BT-20 and MDA-MB-231 (*p* < 0.0001). NSDHL knockdown significantly increased the rate of erlotinib-induced cell death, especially in MDA-MB-231 (*p* = 0.01). NSDHL knockdown led to significantly decreased tumor growth and lung metastasis in the MDA-MB-231 xenograft model (*p* < 0.01). Clinically, high NSDHL expression in tumors of patients with breast cancer was associated with significantly reduced recurrence-free survival (*p* < 0.0001).

**Conclusions:**

NSDHL might have a role in promoting breast cancer progression. The usage of NSDHL as a therapeutic target in breast cancer needs to be clarified in further studies.

## Background

Given that cholesterol is required for cell growth and division, elevated cholesterol levels and abnormal lipid metabolism have been recognized as casual signatures of cancer cells including breast cancer cells and contribute to tumor expansion and malignant progression [1, 2]. Many cancer cells display increased levels of cholesterol biosynthesis genes, which lead to aberrant regulation of cholesterol metabolism [3]. Dietary treatments and drugs targeting high cholesterol synergize with chemotherapeutic agents in decreasing multidrug resistance development [4, 5]. Upregulated cholesterol synthesis is associated with decreased patient survival rates [6]. Recently, high expression levels of cholesterol biosynthesis genes are found to be correlated with poor outcomes in patients with basal-like breast cancer [7]. Therefore, genes involved in cholesterol biosynthesis pathway have become an attractive target in cancer therapy.

NAD(P)-dependent steroid dehydrogenase-like (NSDHL) gene encodes a sterol dehydrogenase or decarboxylase enzyme involved in cholesterol biosynthesis [8]. NSDHL catalyzes the oxidative decarboxylation of the C4 methyl group from meiosis-activating sterol (MAS) and plays a critical role in the synthesis of cholesterol [9]. High NSDHL expression is associated with highly proliferative cells [10]. Downregulation of NSDHL expression results in decreased intracellular cholesterol levels and abrogates the proliferative effect of oncogenic KRAS in fibroblasts [9]. Conditional inactivation of NSDHL antagonizes skin tumor proliferation and prevents the development of pancreatic ductal adenocarcinoma in a mouse model [9, 11]. More intriguingly, knockdown of cholesterol biosynthesis pathway gene NSDHL, markedly sensitizes tumor cells to epithelial growth factor receptor (EGFR) inhibitors [9, 12]. Depletion of NSDHL results in accumulation of MAS, leading to increased EGFR degradation [12] .

The aforementioned studies indicate that NSDHL might play a critical role in malignant tumor progression and could be an unfavorable prognostic factor in cancer patients. Breast cancer is the most commonly diagnosed cancer in women, and female breast cancer ranks as the fifth leading cause of death globally [13]. However, the function of NSDHL in breast cancer cells and in breast cancer progression remains unclear. Therefore, in this study, we investigated the response of breast cancer cells and tumor progression to NSDHL knockdown in breast cancer cells and in xenograft tumor mice. Furthermore, we evaluated the prognostic significance of NSDHL expression in breast cancer patients using data from a public database.

## Methods

### Cell lines and culture

Normal breast epithelial cell line (MCF10A) and human breast cancer cell lines (luminal A: MCF-7, luminal B; ZR-75-1 and BT-474, HER2 amplified: SK-BR-3, basal-like and triple negative (TN): BT-20, and MDA-MB-231) were used in this study. MCF10A(ATCC® CRL-10317™), MCF-7(ATCC® HTB-22), and MDA-MB-231(ATCC® HTB-26) cells were obtained from American Type Culture Collection (Manassas, VA, USA) in 2012–2013. BT-20 (KCLB No. 60061), BT-474 (KCLB No. 60062), SK-BR-3 (KCLB No. 30030), and ZR-75-1 (KCLB No. 21500) cells were obtained from Korean Cell Line Bank (Seoul, Korea) in 2015–2018. Cells were passaged in our laboratory for less than 6 months after thawing frozen aliquots. All cells were authenticated and validated by short-tandem repeat DNA profiling (AmplFLSTR identifiler PCR Amplification kit) and tested to be free of mycoplasma by real-time PCR before use. MCF-7, and MDA-MB-231 cells were grown in DMEM (WelGENE, Seoul, Korea) supplemented with 10% fetal bovine serum (FBS) (WelGENE) and 1% Antibiotic-Antimycotic (Gibco, Carlsbad, CA, USA). BT-20, BT-474, SK-BR-3, and ZR-75-1 cells were grown in RPMI 1640 (WelGENE) supplemented with 10% FBS and 1% antibiotic-antimycotic (Gibco). All cells were maintained at 37 °C in a humidified atmosphere of 95% air and 5% CO_2_.

### Antibodies and drugs

For western blot and immunohistochemistry, we used the following antibodies: β-actin (sc-47778) and sterol regulatory element binding transcription factor 1 (SREBP-1) (sc-365513) from Santa Cruz (Santa Cruz, CS, USA); NSDHL (ab190353) and EGFR (ab52894) from Abcam (Cambridge, UK). For the drug sensitivity test of EGFR tyrosine kinase inhibitor, erlotinib-HCl (OSl-744) (Selleckchem, Houston, TX, USA) was used.

### Small interfering RNA (siRNA) transfection

NSDHL-targeting siRNA (siNSDHL) and scrambled non-targeting control siRNA (siCtrl) were obtained from Dharmacon (Lafayette, CO, USA). The siNSDHL sequence was GAGGAUAUGCUGUCAAUGU and the siCtrl sequence was UGGUUUACAUGUCGACUAA. Transient transfection of cells was performed using Lipofectamine 2000 RNAiMAX Reagent (Thermo Fisher Scientific, Waltham, MA, USA). In brief, 4 × 10^5^ cells were seeded in each well of 12-well plate 24 h prior to transfection. 10 nM and 20 nM siRNA were diluted in Opti-MEM® I Reduced Serum Medium without serum and mixed with Lipofectamine 2000 RNAiMAX Reagent. The mixture was added to each well. The cells were incubated for 24–48 h at 37 °C in a CO_2_ incubator.

### Short hairpin RNA (shRNA) lentiviral transduction

Precisely, 4 × 10^5^ MDA-MB-231 cells were seeded in each well of 12-well plate 24 h prior to viral infection and were replaced with media containing 5 μg/ml polybrene® (sc-134220, Santa Cruz) and 20 μl of NSDHL-targeting shRNA lentiviral particles (shNSDHL) (sc-90849-V, Santa Cruz) or control shRNA lentiviral particles (shCtrl) (sc-108080, Santa Cruz). After 24 h, the transduced cells were selected with 10 μg/ml puromycin dihydrochloride (sc-108071, Santa Cruz) for 7 days.

### Quantitative reverse transcription-polymerase chain reaction (qRT-PCR)

Total RNA was extracted from cells using Tri-RNA Reagent (FAVORGEN, Kaohsiung, Taiwan). qRT-PCR reactions were conducted using cDNA kit (Applied Biosystems, Foster City, CA, USA). Real-time PCR reactions were run on a Light Cycler 480 II (Roche, Salt Lake City, UT, USA) using a SYBR Green PCR master mix (Applied Biosystems) and the specific primers for GAPDH (forward: 5′-GAGTCCAGGGCGTCTTCA-3′, reverse: 5′-GGGGTGCTAAGCAGTTGGT) and NSDHL (forward: 5′-GGTGACGCACAGTGGAAAAC-3′, reverse: 5′-TCGCACGGACTCATTTGACA3’). Results were analyzed using 2^−ΔΔ^CT method [14], which reflects the threshold difference between a target gene and GAPDH in each sample and the relative gene expression, with the reference sample set to 1 (control).

### Western blotting

After the transfection of cells with 10 nM and 20 nM siRNA for 48 h, total cell lysates were collected. Total cell lysates (30–100 μg) were separated using SDS-PAGE and transferred onto Immobilon–P Transfer Membranes (Merck Millipore, Bedford, MA, USA). After blocking with 5% non-fat dry milk in TBS-T or 5% BSA in TBS-T at room temperature for 1 h, the membrane was incubated with primary antibodies (β-actin [1:2000], SREBP-1 [1:500], NSDHL [1:10000], and EGFR [1:10000]) overnight at 4 °C and horseradish peroxidase-conjugated secondary antibodies at room temperature for 30 min and visualized using SuperSignal West Pico Chemiluminescent Substrate (Thermo Fisher Scientific) and Amersham™ Imager 600 (GE Healthcare, Buckinghamshire, UK). The relative intensity of the bands observed in western blotting was analyzed using ImageJ software (National Institutes of Health, Bethesda, MD, USA).

### Cell viability assay

Cells were transfected with 20 nM siRNA for 48 h. Transfected cells were incubated for 1, 2, 3, and 4 days and cell viabilities were evaluated using CellTiter-Glo® Luminescent Cell Viability Assay Kit (Promega, Madison, WI, USA) according to the manufacturer’s instructions. Luminescence was read using SpectraMax 190 Microplate Reader (Molecular Devices, Silicon Valley, CA, USA).

### Cell cycle analysis

Totally, 1 × 10^6^ cells were fixed in 70% cold ethanol overnight at 4 °C. Subsequently, 10 μg/ml propidium iodide (Sigma-Aldrich, St. Louis, USA) was added and cell cycle analysis by quantitation of DNA content was performed using flow cytometry (BD Bioscience, Mansfield, MA, USA). Data were analyzed using ModFit 3.0 (BD Bioscience).

### Colony formation assay

Totally, 5 × 10^3^ cells transfected with 20 nM siRNA for 48 h were seeded in each well of 6-well plate. After day 9, the surface area (μm^2^) of each cell colony was measured. The colonies were fixed with 4% paraformaldehyde and stained with 0.1% crystal violet solution. Crystal violet was then dissolved using a 10% acetic solution, and the absorbance was read using SpectraMax 190 Microplate Reader (Molecular Devices) at 570 nm.

### Three-dimensional Matrigel culture

A layer of growth factor-reduced Matrigel was made for 3D cultures [15]. In brief, 8-well chamber culture plates were coated with a thin layer of 8 mg/ml Matrigel (BD Biosciences) and incubated for 15–30 min at 37 °C to allow the Matrigel to solidify. The mixture of cells (5–6 × 10^6^ cells/ml) and 5 mg/ml Matrigel was added onto the pre-coated surface. After cells were cultured for 9 days, formation of spheroids was observed under a microscope (Leica, Wetzlar, Germany) and the sphere surface area (A = πr^2^) of each spheroid was measured.

### Drug treatment assay

The effect of erlotinib-HCl on siRNA-transfected MDA-MB-231, and BT-20 cells was evaluated using CellTiter-Glo Assay Kit. Totally, 3 × 10^3^ cells were plated in each well of 96-well plate. After 24 h, the complete medium was replaced with various doses (0.04–160 μM) of erlotinib-HCl and the cells were further incubated at 37 °C for 72 h.

### Transwell migration and invasion assays

Cell migration and invasion abilities were assessed using transwell chambers with an 8-μm pore size insert (Costar, Cambridge, MA, USA). For transwell migration assay, cells were transfected with 20 nM siRNAs for 48 h and seeded in the upper chambers at a density of 5 × 10^4^ in serum-free medium and the lower chambers were filled with DMEM containing 10% FBS. After incubation for 24 h at 37 °C, the migrated cells were fixed with 4% paraformaldehyde and stained with 0.1% crystal violet solution.

For invasion assay, the upper chambers were coated with 100 μl of 1 mg/ml Matrigel (BD Biosciences). Cells transfected with 20 nM siRNAs for 48 h were seeded on the Matrigel in the upper chambers at a density of 5 × 10^4^ in serum-free medium and the lower chambers were filled with DMEM with 10% FBS. After incubation for 24 h at 37 °C, the invasive cells were fixed with 4% paraformaldehyde and stained with 0.1% crystal violet solution.

Images of the stained cells were acquired using a microscope equipped with a CCD camera (Leica). Crystal violet was then extracted with a 10% acetic acid solution, and absorbance was read using SpectraMax 190 Microplate Reader (Molecular Devices) at 570 nm.

### Wound healing assay

After cells were transfected with 20 nM siRNAs for 48 h, 4 × 10^5^ cells were seeded in each well of 24-well plate in triplicates and incubated at 37 °C overnight. After the cells reached 100% confluence to form a monolayer, a scratch of the cell monolayer was created using a pipette tip and the cells were incubated for 24 h. The first image (0 h) and closure image (24 h) of the scratch was acquired using a microscope equipped with a CCD camera (Leica) and the area to close the wound was measured.

### Total cholesterol assay

Total intracellular cholesterol level in cells was measured using Total Cholesterol Assay Kit (Fluorometric) (Cell Biolab, Inc., San Diego, CA, USA) [16]. Cholesterol standards were prepared according to the manufacturer’s instructions to construct the calibration curve of cholesterol. In brief, cells transfected with 20 nM siRNA for 48 h were washed with cold phosphate-buffered saline. Total cholesterol was extracted from 1 × 10^6^ cells using 200 μl of a mixture of chloroform:isopropanol: NP-40 (7:11:0.1). The extracts were transferred to a new tube and dried at 50 °C to remove the chloroform and then put under vacuum for 30 min to remove the trace amounts of organic solvent. Dried lipids were dissolved in 200 μl of 1X Assay Diluent with sonicating and vortexing until the solution is homogenous. An amount of 25 μl of diluted cholesterol standards/serum samples and 25 μl of the cholesterol reaction reagent containing cholesterol esterase and cholesterol oxidase, fluorometric probe, and horseradish peroxidase were added to each well. After incubation for 45 min at 37 °C, the concentration of cholesterol within samples was immediately measured with the fluorescence microplate reader (Synergy H1, BioTek Instruments, Inc., Winooski, VT, USA) at excitation of 550 nm and emission of 595 nm.

### Xenograft animal model

NOD.Cg-Prkdc^scid^ Il2rg^tm1wjl^ /SzJ mice (NSG mice) were obtained from The Jackson Laboratory (Bar Harbor, ME, USA). All animal experiments were approved by the Seoul National University Institutional Animal Care and Use Committee (IACUC, SNU 15112–3-4). A total of 10 female NSG mice were used. Orthotropic xenografts were established via injection of 1 × 10^6^ shNSDHL MDA-MB-231 cells (*n* = 5) or shControl cells (*n* = 5) mixed with Matrigel (BD Biosciences) into the fat pad of the 4th mammary gland of 5-week old mice. After injection of tumor cells, primary tumor volume was measured weekly using digital calipers and a modified ellipsoidal formula (volume = 1/2(length×width^2^)) [17].

### Immunohistochemistry

Tissues were fixed with 10% buffered formalin and embedded in paraffin blocks. Blocks of paraffin-embedded tissues were sectioned into 4 μm sections. For immunohistochemistry (IHC), the sections were deparaffinized in xylene, rehydrated in a graded series of ethanol (100%, 90%, and 75%), and pretreated with autoclaving at 98 °C for 20 min in 10 mM Tris/1 mM EDTA (pH 9.0) for antigen retrieval. Endogenous peroxidase activity was blocked by incubation with 3% H_2_O_2_ for 30 min at room temperature. The sections were incubated with 10% normal goat serum for 1 h to block nonspecific binding of immunological reagents. After incubation with primary antibodies for NSDHL (1:500) at 4 °C overnight, horseradish peroxidase-conjugated secondary antibodies were applied, and reaction products were visualized using the DAB chromogen kit (Agilent Technologies, Produktionsvej, Glostrup, Denmark) and counterstained with hematoxylin solution (Merck Millipore) according to the manufacturer’s instructions. Histological images of stained tissues were acquired using a microscope equipped with a CCD camera (Leica). Five fields at 40× magnification within each section were randomly selected, and the immunostained area was quantified as the percentage of NSDHL-positive area in each field by QWin image-analysis and image-processing software (Leica).

For the analysis of lung metastasis from hematoxylin and eosin (H&E) staining, six fields at 40× magnification within one central section of tumor tissue per animal were randomly selected and metastatic foci were quantified using ImageJ software (National Institutes of Health).

### Clinical prognostic implication of NSDHL expression level in the survival of breast Cancer patients

From the Gene expression omnibus (GEO) database [18], we downloaded clinical information and microarray gene expression profiles for 3951 patients with breast cancer including patients with luminal A (*n* = 1933), luminal B (*n* = 1149), HER2-positive (*n* = 252), HER2-negative (*n* = 800), basal-like (*n* = 618), ER-positive (*n* = 3083), ER-negative (*n* = 873), and TN (*n* = 198) subtypes. Recurrence-free survival (RFS) analyses were performed using Kaplan-Meier method. The log-rank *p*-value was used to identify differences in RFS. Cox proportional hazard regression models were used to estimate hazard ratios (HR) with 95% confidence intervals (CI). A multivariate Cox regression model was fitted based on all characteristics that had *p* < 0.05 in the univariate analysis. HR of greater than one indicates that the marker was associated with poor prognosis, while a ratio of less than one means that it was associated with good prognosis.

### Statistics

In the analysis of data obtained in vitro and in vivo, graphs were represented as mean ± standard deviation of at least three independent experiments. The statistical comparisons between the two independent groups were made using unpaired t-test. For groups of three or more, data were analyzed with Kruskal-Wallis nonparametric ANOVA followed by the Dunn’s multiple comparison test. Statistical analyses were performed by GraphPad Prism v6.01 (GraphPad Software Inc., La Jolla, CA, USA). For all tests, a *p*-value less than 0.05 was considered statistically significant.

## Results

### NSDHL protein level was higher in BT-20 and MDA-MB-231 cells than in the other breast cancer cells and normal epithelial cells

**Fig. 1:**
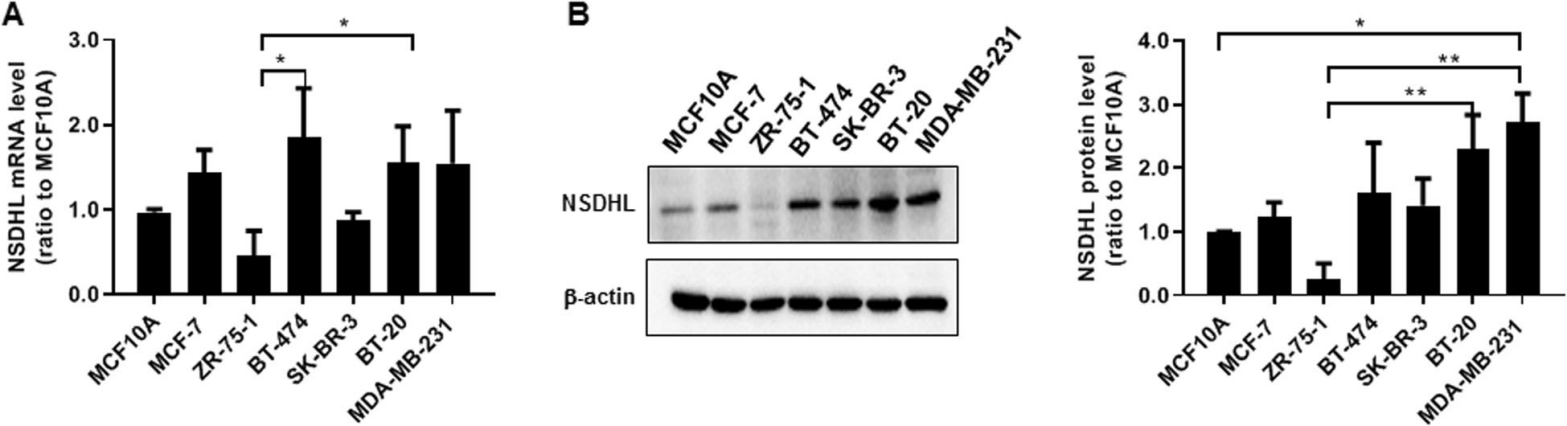
BT-20 and MDA-MB-231 cells display higher levels of NSDHL expression compared to those of other breast cancer cells and normal breast normal breast epithelial cell. **a, b** Data of relative NSDHL mRNA and protein levels analyzed by real-time RT-PCR and western blot in breast cancer cell lines (MCF-7, ZR-75-1, BT-474, SK-BR-3, BT-20, MDA-MB-231) and normal breast epithelial cell line (MCF10A); Data represent the means ± standard deviations of four independent experiments

The NSDHL mRNA and protein levels were evaluated in six human breast cancer cell lines (MCF-7, ZR-75-1, BT-474, SK-BR-3, BT-20, and MDA-MB-231) and a human epithelial cell line (MCF10A). The relative levels of NSDHL mRNA were increased in MCF-7, BT-474, BT-20 and MDA-MB-231 compared to those of MCF10A and the other breast cancer cells (*n* = 4) (Fig. 1a). NSDHL protein level was higher in BT-20 and MDA-MB-231 than in the other breast cancer cells and MCF10A (*n* = 4) (Fig. 1b). ZR-75-1 expressed less NSDHL mRNA and protein than the MCF10A. Multiple comparison analysis (the Kruskal-Wallis test followed by the Dunn’s test) of NSDHL mRNA levels showed a significant difference among ZR-75-1, BT-474 and BT-20 (ZR-75-1 vs. BT-474, *p* = 0.025; ZR-75-1 vs. BT-20, *p* = 0.024). In multiple comparison analysis of NSDHL protein levels, there was a significant different among MCF10A, ZR-75-1, BT-20 and MDA-MB-231 (MCF10A vs. MDA-MB-231, *p* = 0.034; ZR-75-1 vs. BT-20, *p* = 0.0098; ZR-75-1 vs. MDA-MB-231, *p* = 0.0015).

### NSDHL knockdown decreased viability and proliferation of breast cancer cells

To examine the role of NSDHL in the proliferation and migration of cancer cells, NSDHL-knockdown cells were produced by transfection of siRNAs into MCF-7, MDA-MB-231, and BT-20 cells. The transfection of NSDHL siRNA (10 nM and 20 nM) significantly downregulated NSDHL mRNA and protein expressions in MCF-7, MDA-MB-231, and BT-20 cells (*n* = 3, *p* < 0.01 and *p* < 0.001) (Fig. S1A-F).

**Fig. 2:**
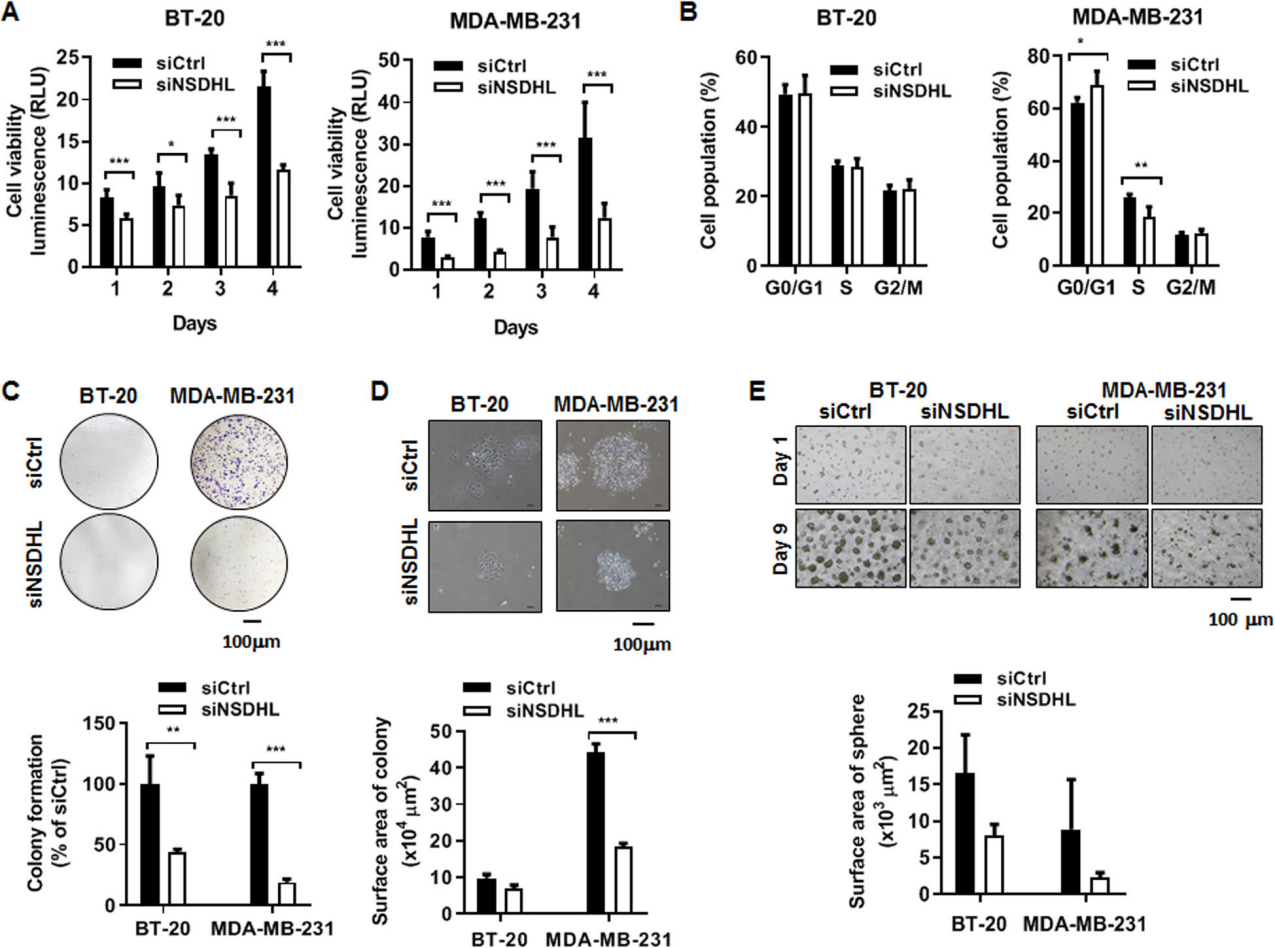
NSDHL knockdown decreases viability, proliferation, and colony and sphere formation abilities of BT-20 and MDA-MB-231 cells. **a, b** Data of cell viability and cell cycle in BT-20 and MDA-MB-231 cells transfected with NSDHL siRNA or control siRNA (20 nM); **c, d** Representative images and data analyzed in colony formation of BT-20 and MDA-MB-231 cells transfected with NSDHL siRNA or control siRNA (20 nM); **e** Representative image and data analyzed in 3D sphere formation of BT-20 and MDA-MB-231 cells transfected with NSDHL siRNA or control siRNA (20 nM). All data represent the means ± standard deviations of three independent experiments, each performed in triplicates. *p < 0.05, **p < 0.01, ***p < 0.001

In CellTiter-Glo® Luminescent Cell Viability Assay, siRNA-mediated NSDHL-knockdown BT-20 and MDA-MB-231 cells revealed up to 50–70% drop in cell viability (*n* = 3, *p* < 0.05, *p* < 0.01 and *p* < 0.001) (Fig. 2a). In cell cycle analysis of MDA-MB-231 cells, NSDHL knockdown resulted in a significant increase in the proportion of G0/G1 cells (siNSDHL vs. siCtrl: 69.15 ± 5.16% vs. 62.32 ± 1.93%, *n* = 3, *p* = 0.012) and a significant decrease in the proportion of S cells (siNSDHL vs. siCtrl: 18.63 ± 3.7% vs. 25.79 ± 1.46%, *n* = 3, *p* = 0.002) (Fig. 2b). However, no significant cell cycle difference was observed in NSDHL-knockdown BT-20 and control BT-20 (*n* = 4) (Fig. 2b). Correlated with the results of cell viability and cell cycle analysis, NSDHL knockdown significantly suppressed colony formation (siNSDHL vs. siCtrl: 19.08 ± 2.67% vs. 100.00 ± 8.56%, *n* = 3, *p* < 0.001) and surface area (siNSDHL vs. siCtrl: 18.23 ± 0.96 μm^2^ vs. 44.08 ± 2.41 μm^2^, *n* = 3, *p* < 0.001) in MDA-MB-231 cells (Fig. 2c-d). However, there was no significant decrease in 3D sphere formation (siNSDHL vs. siCtrl: 2.31 ± 0.63 μm^2^ vs. 8.84 ± 6.85 μm^2^, *n* = 3, *p* = 0.175) in MDA-MB-231 cells (Fig. 2e). Although NSDHL knockdown in BT-20 cells caused a decrease in colony formation, surface area, and 3D sphere formation, there was a significant difference in only colony formation between control and knockdown cells (siNSDHL vs siCtrl: 43.24 ± 2.87 vs 100 ± 23.06, *n* = 3, *p* = 0.013) (Fig. 2c-e). The inhibitory effect of NSDHL knockdown on cell growth, cell cycle, cell colony, and 3D sphere formation was greater in MDA-MB-231 cells than in BT-20 cells. In NSDHL-knockdown MCF-7 cells, a significant inhibitory effect on cell growth, cell colony, and 3D sphere formation and G0/G1 phase arrest were observed (*n* = 4) (Fig. S2A-E).

### NSDHL knockdown inhibits the migration and invasion abilities of breast cancer cells

**Fig. 3:**
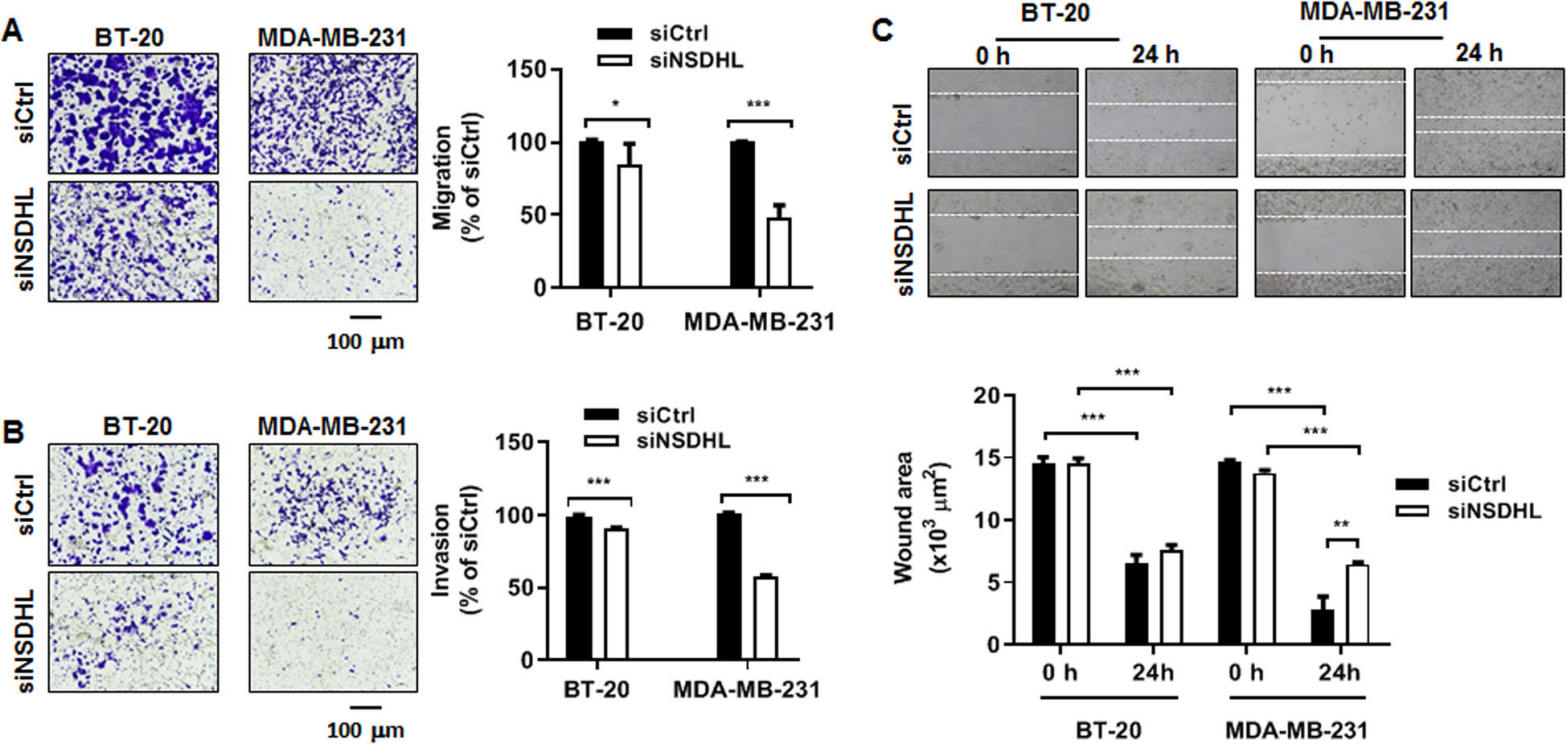
NSDHL knockdown decreases the migration and invasion abilities of BT-20 and MDA-MB-231 cells. **a,b,c** Representative images and data analyzed in transwell migration assay, invasion assay and, wound healing assay of BT-20 and MDA-MB-231 cells transfected with NSDHL siRNA or control siRNA (20 nM). Data represent the means ± standard deviations of three independent experiments. *p < 0.05, **p < 0.01, ***p < 0.001

To examine the role of NSDHL in breast cancer cell migration and invasion, transwell migration, invasion, and wound healing assays were performed. An FBS gradient (0% FBS on top and 10% FBS on bottom) was made to induce migration. Transwell migration assay showed that NSDHL knockdown significantly suppressed the migration ability of MDA-MB-231 (siNSDHL vs. siCtrl: 47.85 ± 8.65% vs. 100.1 ± 0.47%, *n* = 3, *p* < 0.001) and BT-20 cells (siNSDHL vs. siCtrl: 84.26 ± 14.54% vs. 100.40 ± 1.21%, *n* = 3, *p* = 0.022) (Fig. 3a). The Matrigel-based invasion assay performed to measure the invasion of cells through extracellular matrix revealed that NSDHL knockdown led to a significant suppression in the invasion capacity of MDA-MB-231 (siNSDHL vs. siCtrl: 57.80 ± 0.49% vs. 100.55 ± 1.0%, *n* = 3, *p* < 0.001) and BT-20 cells (siNSDHL vs. siCtrl: 91.03 ± 0.17% vs. 99.26 ± 0.95%, *n* = 3, *p* < 0.001) (Fig. 3b). Likewise, wound healing assay showed that NSDHL knockdown resulted in a significant decrease in the collective migration of MDA-MB-231 cells (siNSDHL vs. siCtrl: 2.80 ± 1.06 μm^2^ vs. 6.47 ± 0.15 μm^2^, *p =* 0.004) (Fig. 3c). BT-20 cells did not exhibit a significant decrease in wound healing assay (siNSDHL vs. siCtrl: 6.5 ± 0.71 μm^2^ vs. 7.6 ± 0.4 μm^2^, *p* = 0.105) (Fig. 3c). In MCF-7 cells, NSDHL knockdown resulted in a significant decrease in wound healing and transwell migration activities (Fig. S3A and S3B). In Matrigel-based invasion assay, an invasion activity in both NSDHL knockdown MCF-7 cells and control MCF-7 cells was not observed.

### NSDHL knockdown reduced the amount of cellular cholesterol and sensitized the breast cancer cells to the cytotoxic effects of erlotinib

**Fig. 4:**
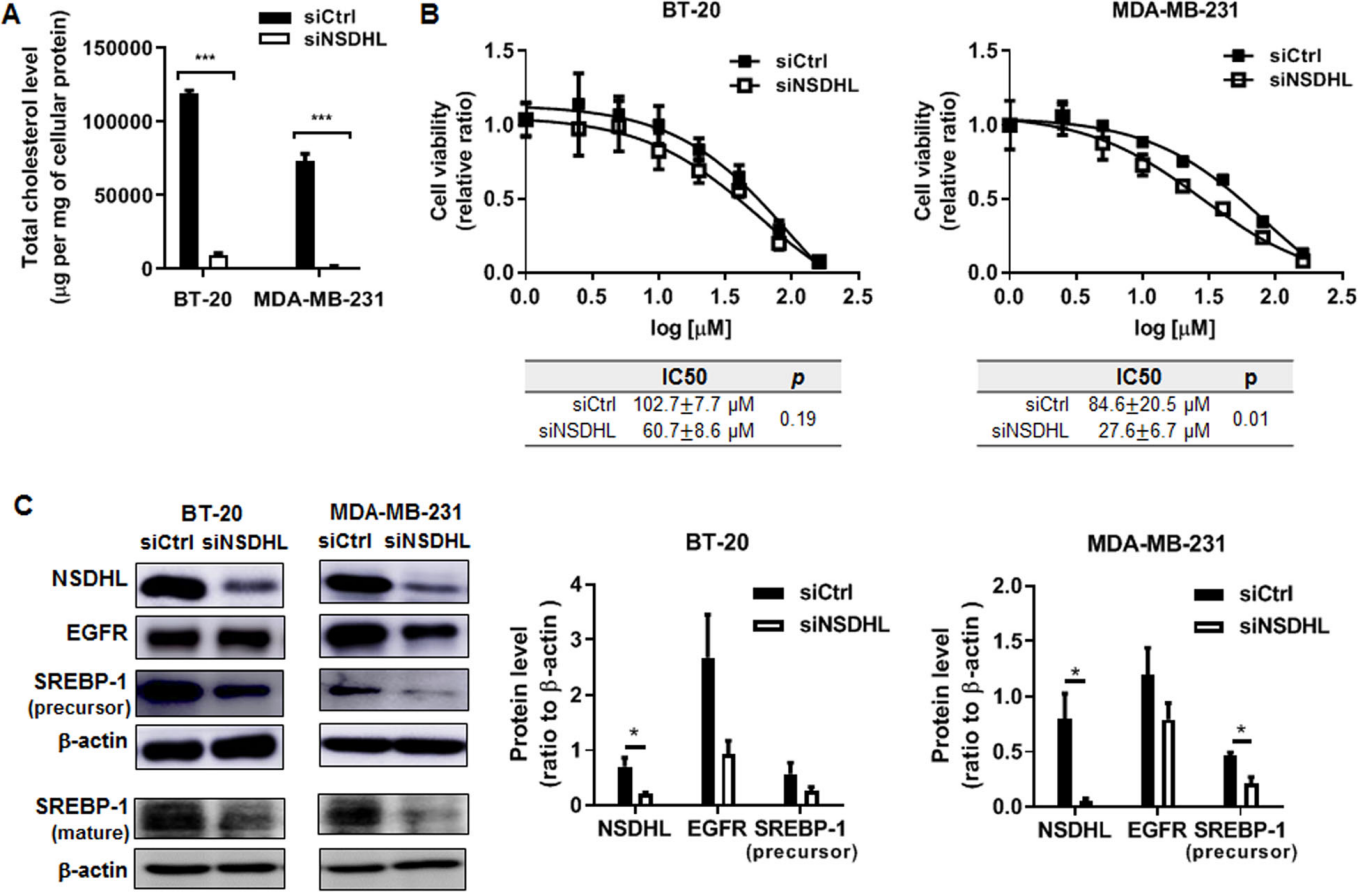
NSDHL knockdown suppresses total cholesterol level and promotes erlotinib response in MDA-MB-231 cell. **a** Total cholesterol levels measured in BT-20 and MDA-MB-231 cells transfected with NSDHL siRNA or control siRNA (20 nM); **b** Dose-response curve of *erlotinib* in BT-20 and MDA-MB-231 cells transfected with NSDHL siRNA or control siRNA (20 nM); **c** Representative western blot images of NSDHL, EGFR, and precursor and mature SREBP-1 and data of relative expression levels of NSDHL, EGFR, and precursor SREBP-1 in BT-20 and MDA-MB-231 cells transfected with NSDHL siRNA or control siRNA (20 nM). Data represent the mean ± standard deviation of three independent experiments. *p < 0.05, ***p < 0.001

To investigate whether NSDHL knockdown can cause a reduction in the amount of cellular cholesterol, total intracellular cholesterol content was measured from the extract of breast cancer cells. As compared with those of control cells, NSDHL knockdown resulted in a significant decrease in total intracellular cholesterol content of BT-20 cells (siCtrl vs siNSDHL; 118,899.5 ± 2412.6 μg/mg of cellular protein vs 9445.4 ± 1395.1 μg/mg of cellular protein, *p* < 0.0001) and MDA-MB-231 cells (siCtrl vs siNSDHL; 72,951.5 ± 5350.4 μg/mg of cellular protein vs 1436.5 ± 706.9 μg/mg of cellular protein, *p* < 0.0001), respectively (Fig. 4a).

To investigate whether NSDHL gene expression is involved in the sensitization effect to EGFR inhibitor, half inhibitory concentration (IC50) of erlotinib was calculated using CellTiter-Glo® Luminescent Cell Viability Assay Kit. Erlotinib decreased cell viability in a dose-dependent manner in breast cancer cells MB-231, BT-20, and MCF7 as well as normal breast epithelial cell, MCF10A cells. There was no significant difference in IC50 values between NSDHL-knockdown BT-20 and control cells (siCtrl vs. siNSDHL: 102.7 ± 7.7 μM vs. 60.7 ± 8.6 μM, *n* = 3, *p* = 0.19). In MCF-7 and MCF10A cells, a synergistic effect of NSDHL knockdown with erlotinib was not observed (Fig. S4A and S4B). However, IC50 values in NSDHL-knockdown MDA-MB-231 were significantly lower than that of control cells (siCtrl vs. siNSDHL: 84.6 ± 20.5 μM vs. 27.6 ± 6.7 μM, *n* = 3, *p* = 0.01), indicating that NSDHL knockdown specifically synergized with erlotinib in MDA-MB-231 cells (Fig. 4b).

Accumulation of MAS through the inactivation of NSDHL reduces EGFR expression [12]. SREBP-1a and SREBP-1c regulate expression of genes involved in cholesterol but also in other lipid metabolism [19]. We explored whether NSDHL is involved in the regulation of EGFR, and SREBP-1. Interestingly, NSDHL knockdown downregulated EGFR and precursor and mature forms of SREBP-1 in both MDA-MB-231 and BT-20 cells (Fig. 4c). However. NSDHL knockdown was not associated with a significant decrease in EGFR neither in MDA-MB-231 cells nor in BT-20 cells. Densitometry analysis showed the precursor form of SREBP-1 in only NSDHL-knockdown MDA-MB-231 was significantly downregulated as compared with those of control cells (siCtrl vs. siNSDHL: 0.47 ± 0.05 vs. 0.22 ± 0.09, *n* = 3, *p* = 0.015).

### NSDHL knockdown decreased breast tumor growth and lung metastasis

**Fig. 5:**
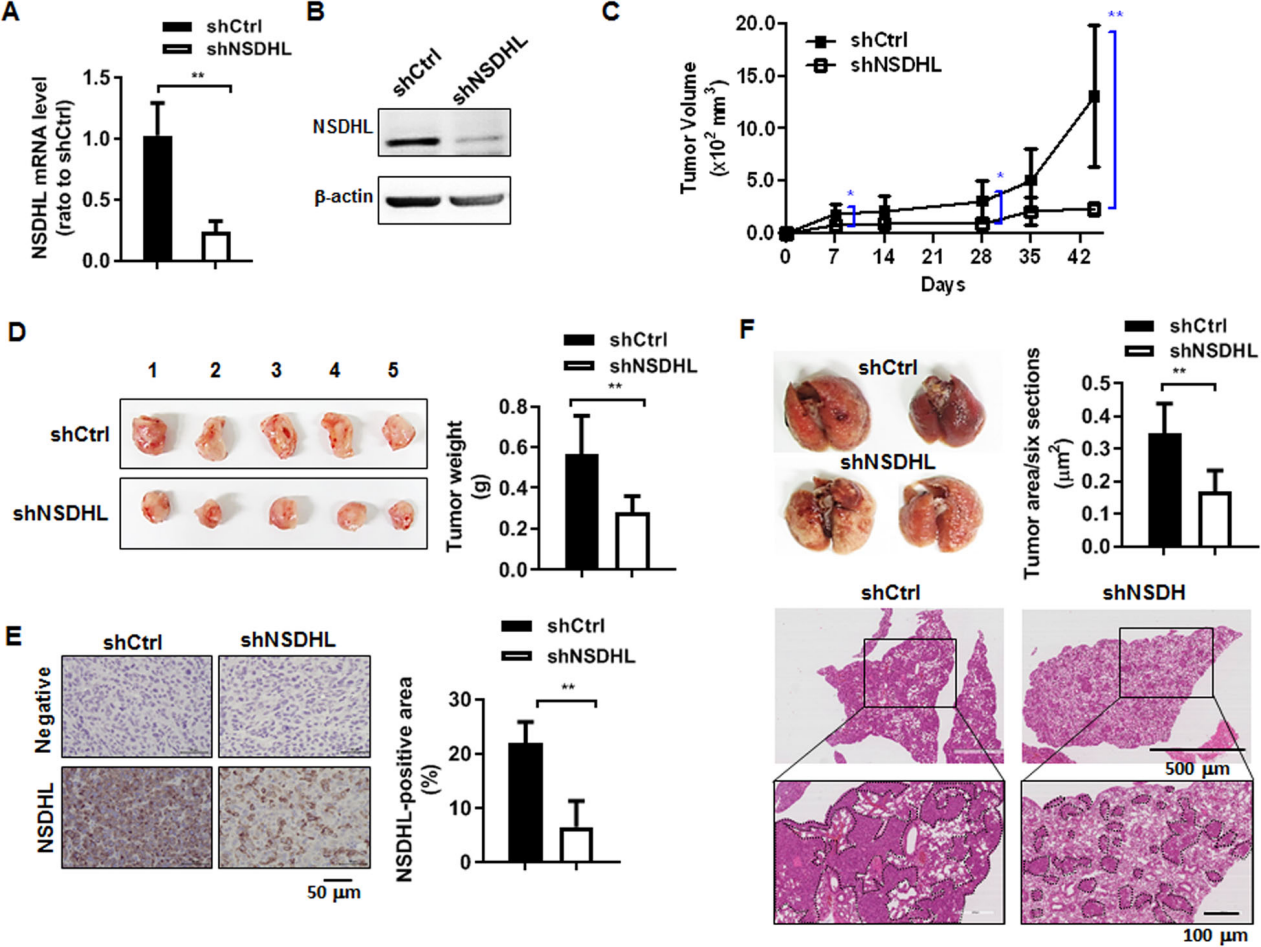
NSDHL knockdown suppressed tumor growth and lung metastasis of MDA-MB-231 xenograft mice. **a, b** Data of relative NSDHL mRNA level analyzed by real-time RT-PCR and representative western blot images of NSDHL in MDA-MB-231 cells transduced with NSDHL shRNA or control shRNA lentivirus; **c** Data of tumor volume measured weekly in NSDHL shRNA or control shRNA mice; **d** Gross images and wet weight of tumors removed from NSDHL shRNA or control shRNA mice at 44 days post-injection; **e** Representative NSDHL immunohistochemistry image and scores analyzed from NSDHL shRNA or control shRNA tumor tissues; **f** Gross and H&E images of lungs and data of metastatic foci analyzed from NSDHL shRNA or control shRNA lung tissues. In vitro data represent the means ± standard deviations of three independent experiments. Animal data represent the means ± standard deviations of five mice per group. *p < 0.05, **p < 0.01

To explore the role of NSDHL in breast tumor growth and metastasis, orthotropic breast cancer models were created by injection of MDA-MB-231 cells transduced with lentivirus-carrying shRNA-targeting NSDHL. It has been shown in Fig. 5a and b that NSDHL mRNA and protein were downregulated in NSDHL shRNA-transduced MDA-MB-231 cells. Primary tumor volumes measured weekly were significantly lower in the NSDHL knockdown group (*n* = 5) than in the control group (*n* = 5) (Fig. 5c). As shown in the gross images of the tumors excised at the end of 44 days, tumor wet weights were significantly lower in the NSDHL knockdown group (*n* = 5) than in the control group (*n* = 5) (Fig. 5d).

The representative IHC for NSDHL showed strong staining in the control tumor and overall weak staining in the NSDHL-knockdown tumor. The NSDHL-stained cells in NSDHL-knockdown tumor tissue may be the tumor cells not to completely knockdown the expression of a NSDHL gene by shRNA and stromal cells. Quantitative analysis of IHC revealed that NSDHL-positively stained cells and area were significantly reduced in the NSDHL-knockdown tumor (*n* = 5, 6.53 ± 4.76%) than in the control tumor (*n* = 5, 22.00 ± 3.89%) (Fig. 5e, *p* = 0.005). In the gross image of the excised lung and the sectioning of the lung followed by H&E staining, heavy tumor burden was detected in the shNSDHL-knockdown group (Fig. 5f). The metastatic area in the lung was significantly reduced in the shNSDHL-knockdown group (*n* = 5, 0.17 ± 0.06%) than in the control group (*n* = 5, 0.35 ± 0.09%) (Fig. 5f, *p* = 0.004).

### High expression of NSDHL is associated with reduced survival in patients with breast cancer

**Fig. 6:**
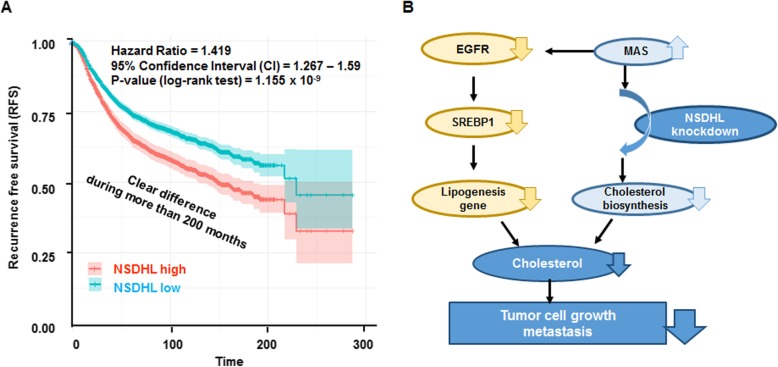
NSDHL expression correlates with poor prognosis in breast cancer. **a** Kaplan-Meier plots of recurrent free survival based on the combination of NSDHL expression in breast cancer patient; **b** Schematic overview of the regulatory mechanisms affected by NSDHL knockdown in breast tumor growth and metastasis. Knockdown of NSDHL may result in an accumulation of meiosis-activating sterol (MAS), which influences EGFR degradation, and dysregulated EGFR-mediated signals suppresses SREBP-1 expression, leading to suppression of lipogenesis and cholesterol biosynthesis

To analyze the clinical significance of NSDHL expression in patients with breast cancer, survival analysis in 3951 breast cancer patients was performed using public database. In univariate analysis, patients with high NSDHL expression had reduced RFS compared to those with lower expression (HR: 1.419, 95% CI: 1.267–1.59, *p* < 0.001) (Fig. 6a). Detailed information on the univariate and multivariate analysis among NSDHL expression, RFS, and clinicopathological features is shown in Table 1. In subtype analysis, luminal A (HR: 1.30, 95% CI: 1.10–1.55, *p* < 0.002), luminal B (HR =1.37, 95% CI = 1.12–1.68, *p* < 0.002), HER2-amplified (HR: 0.83, 95% CI: 0.56–1.24, *p* < 0.372) and basal-like (HR: 1.37, 95% CI: 1.05–1.78, *p* < 0.002) were significant, but TN (HR: 1.61, 95% CI: 0.91–2.81, *p* < 0.097) was not significant. In tumor grade analysis, tumor grade I (HR: 1.81, 95% CI: 1.08–3.06, *p* < 0.023), tumor grade II (HR: 1.37, 95% CI: 1.07–1.75, *p* < 0.013), and tumor grade III (HR: 0.77, 95% CI: 0.62–0.97, *p* < 0.023) were significant. In a multivariate analysis, the classification of subtypes (luminal A, luminal B, HER2-positive, TN and basal-like) (HR: 1.27, 95% CI: 1.14–1.41, *p* < 0.001), the lymph node (LN) status (LN positive and negative) (HR: 1.33, 95% CI: 1.16–1.153, *p* = 0.000), ER positive and negative status (HR: 1.34, 95% CI: 1.19–1.51, *p* = 0.000) and PR positive and negative status (HR: 1.54, 95% CI: 1.22–1.94, *p* < 0.001) were found to be important predictors of poor RFS (Table 1). However, HER2 positive and negative status (HR: 1.20, 95% CI: 0.93–1.56, *p* < 0.090) was not a significant predictor of RFS in patients with breast cancer.

**Table 1:** Univariate and multivariable analyses with respect to NSDHL

Variables	Univariate			Multivariate		
	HR	95% CI	***p***	HR	95% CI	***p***
Total	1.419	1.267–1.59	< 0.001	–	–	–
Subtype						
Luminal A	1.30	1.10–1.55	< 0.002	–	–	–
Luminal B	1.37	1.12–1.68	< 0.002	–	–	–
HER2	0.83	0.56–1.24	< 0.372	–	–	–
TN	1.61	0.91–2.81	< 0.097	–	–	–
Basal-like	1.37	1.05–1.78	< 0.002	1.27	1.14–1.41	< 0.001
Tumor grade						
I	1.81	1.08–3.06	< 0.023	–	–	–
II	1.37	1.07–1.75	< 0.013	–	–	–
III	0.77	0.62–0.97	< 0.023	–	–	–
LN						
Positive	1.25	1.04–1.50	< 0.015	1.33	1.16–1.53	0.0
Negative	1.42	1.15–1.77	< 0.001	–	–	–
ER						
Positive	1.37	1.20–1.57	< 0.001	1.34	1.19–1.51	0.0
Negative	1.29	1.03–1.61	< 0.028	–	–	–
PR						
Positive	2.03	1.43–2.90	< 0.001	1.54	1.22–1.94	< 0.001
Negative	1.28	0.94–1.73	< 0.115	–	–	–
HER2						
Positive	0.58	0.36–0.92	< 0.019	1.20	0.93–1.56	< 0.090
Negative	1.53	1.16–2.03	< 0.003	–	–	–

The Cox proportional hazards model was used to identify the factors that had a significant influence on recurrence-free survival. *p* < 0.05 was considered as a statistically significant difference. *HR* Hazard Ratio, *CI* Confidential Interval, *LN* Lymph Node, *ER* Estrogen Receptor,* PR* Progesterone Receptor, *HER-2* Human Epidermal Growth Factor 2, *TN* Triple Negative

## Discussion

Cholesterol biosynthesis pathway is commonly elevated or dysregulated in cancer cells and high cholesterol levels are associated with cancer progression [6, 9]. High expression levels of cholesterol biosynthesis genes and high cholesterol levels are associated with increased risks of breast cancer [20]. NSDHL involved in the endogenous pathway of cholesterol biosynthesis has been suggested as a critical target for cancer therapy [9, 21]. However, the role of NSDHL in the biological function of breast cancer cells and its clinical significance in patients with breast cancer are yet to be fully elucidated. In this study, we demonstrated that NSDHL knockdown affects the cell cycle, survival, proliferation, and migration of breast cancer cells, resulting in suppression of breast tumor progression and metastasis. Additionally, our study suggests that high NSDHL expression is a potential predictor of poor prognosis in breast cancer patients.

Cholesterol biosynthesis genes, including NSDHL, sterol C4-methyl oxidase-like (SC4MOL), farnesyl-diphosphate farnesyltransferase 1 (FDFT1), 3-hydroxy-3-methylglutaryl-CoA synthase 1 (HMGCS1), 3-hydroxy-3-methylglutaryl-CoA reductase (HMGCR), emopamil-binding protein (EBP), and 7-dehydrocholesterol reductase (DHCR7) are highly expressed in breast cancer cells [22]. ZR-75-1 cells in breast cancer cell group expressed less NSDHL mRNA and protein than the MCF10A cell line. We observed high level of NSDHL protein expression in basal-like and TN subtype (BT-20 and MDA-MB-231) compared to the other subtypes and normal epithelial cells, suggesting that increased NSDHL expression may be associated with greater cell survival of these breast cancer cell lines. NSDHL is upregulated in highly proliferative cells [10] and inactivation of NSDHL blocks the growth of skin and pancreatic cancer cells [9, 11]. Likewise, we observed that NSDHL knockdown decreased cell viability, colony formation, and 3D sphere formation in MCF-7, MDA-MB-231, and BT-20 cells. The aforementioned studies and our results show compelling evidence for the pivotal role of NSDHL in promoting the survival and proliferation of breast cancer cells. Recently, Ehmsen et al. reported that DHCR7, LSS, FDFT1, EBP, NSDHL, and HMGCS1 directly involved in the enzymatic catalytic steps and CYB5R3 functions as a reductase enzyme in the ER membrane were elevated in mammospheres to reveal stem like features, and suggested the cholesterol biosynthesis pathway is associated with breast cancer stem cell propagation [7]. Further research is required to elucidate the role of NSDHL in breast cancer stem cell propagation.

Cellular cholesterol regulates cell cycle progression by directly influencing the function of membrane proteins involved in cell cycle regulation. *Cholesterol biosynthesis* inhibitors, lovastatin, AY 9944, and triparanol contribute to G1 arrest of cell cycles [23]. In our study, NSDHL knockdown caused reduction of total cholesterol in BT-20 and MDA-MB-231 cells. Especially, cholesterol-lowering effect of NSDHL knockdown was greater in MDA-MB-231 cells, indicating that NSDHL might be largely involved in cholesterol synthesis pathway in MDA-MB-231 cells. We found that a significant increase in S phase and cell cycle arrest in G0/G1 phase in MDA-MB-231 cells was caused by NSDHL knockdown, suggesting that NSDHL knockdown may mediate cell cycle arrest by lowing cholesterol levels.

The transwell migration and Matrigel invasion assays have been used to measure the chemotactic capability of breast cancer cells toward attractants by mimicking the process of extracellular matrix invasion and extravasation commonly found in cancer metastasis. In this study, the transwell migration and invasion abilities of MDA-MB-231 and BT-20 cells were remarkably suppressed by NSDHL knockdown. However, in wound healing assay evaluating the type of collective migration of proliferative cells, NSDHL knockdown did not impair the wound healing function of BT-20 cells effectively as compared to that of MDA-MB-231 cells. This result is in part due to the capacity of BT-20 cells to escape cell cycle arrest under NSDHL knockdown. Given these results, the NSDHL may play a more important role in migration and invasion activities in MDA-MB-231 cells than BT-20 cells.

High EGFR expression is found mainly in the basal-like and TN subtypes of breast cancer [24]. EGFR inhibitors are considered to be drug targets for treatment of basal-like and TN subtypes. However, EGFR-targeted drugs such as erlotinib, cetuximab, lapatinib, and gefitinib did not yield satisfactory results in patients with breast cancer [25]. Inactivation of SC4MOL and NSDHL sensitizes tumor cells to EGFR inhibitors via increased EGFR degradation [12]. Use of cholesterol-lowering drug statins, known as endogenous cholesterol synthesis inhibitors targeting 3-hydroxy-3-methylglutaryl coenzyme-A (HMG-CoA) has been shown to exert several beneficial anti-neoplastic properties, including decreased tumor growth, angiogenesis, and metastasis [26]. Statins have radio-sensitizing activity in pre-clinical models especially in breast cancer stem-like cells [27, 28], and improve survival among patients with breast cancer [29, 30]. Long-term usage of stains enhances the therapeutic effect of EGFR-targeting drugs gefitinib and erlotinib in lung cancer patients [31]. Our data showed the additive and synergistic interaction between erlotinib and NSDHL knockdown in MDA-MB-231 cells, when compared with the other breast cancer cells. In our understanding, the varied patterns of in vitro results in the NSDHL-knockdown breast cell lines may be caused by cell-type specificity. Further studies to elucidate the molecular mechanisms underlying the breast cancer cell-type specific effect of NSDHL knockdown on cell death, cell proliferation, cell migration, cell invasion, cell cycle arrest, and drug response are required.

NSDHL deficiency abrogates the skin tumor development induced by oncogenic KRASG^12D^ in mice [9], implicating that it is involved in tumor development. We observed that NSDHL knockdown suppressed the primary tumor growth as well as lung metastasis in orthotropic breast tumor model injected with NSDHL-knockdown MDA-MB-231 cells. Our in vivo results demonstrate that NSDHL has a significant function in breast cancer progression.

SREBP-1, which activates the genes involved in cholesterol synthesis, promotes migration and invasion in breast cancer [32]. LDLR mediates the cellular uptake of cholesterol [33] and elevated LDLR expression in tumor accelerates LDL cholesterol-mediated breast cancer growth in mouse models [34]. Consistent with these findings, our study shows that NSDHL knockdown caused a significant reduction of total cholesterol level in breast cancer cells, accompanying the downregulation of EGFR and SREBP1, and has an additive or synergistic anti-cancer effect on erlotinib-treated breast cancer cells. Given the “Moonlighting” function of cholesterol pathway enzymes and metabolites [2], we speculate that NSDHL knockdown may result in an accumulation of MAS, which influences EGFR degradation, and dysregulated EGFR-mediated signals suppresses SREBP1 expression, leading to the suppression of lipogenesis, cholesterol biosynthesis, and breast tumor growth and metastasis (Fig. 6b). The inhibitors and gene knockdown associated with cholesterol synthesis may have a collective benefit in combination with EGFR-targeting therapy in patients with breast cancer.

High cholesterol level is implicated as a risk factor and as a poor prognostic factor in breast cancer [7, 35]. According to relapse-free survival analysis reported by Ehmsen S et al. high expression of cholesterol biosynthesis genes NSDHL, EBP, HMGCS1, farnesyl diphosphate synthase (FDPS), FDFT1 is associated with worse prognosis in basal-like subtype of breast cancer patients [7]. In our findings, high NSDHL expression is linked to a decreased RFS in hormone receptor positive (ER/PR-positive) and basal-like subtypes, but there was no significant correlation to predict poorer outcome in HER2 positive subtypes. Furthermore, high level of NSDHL provides additional prognostic information to predict LN metastasis. Thus, our results indicate that high level of NSDHL is a predictive prognostic factor that reduces survival in patients with hormone receptor positive and basal-like subtypes and LN metastasis.

## Conclusions

NSDHL has a crucial role in regulating survival, proliferation, cell cycle, migration and invasion of breast cancer cells and promoting breast cancer progression and metastasis. Clinically, NSDHL is a poor prognostic marker that predicts survival in patients with breast cancer. NSDHL-cholesterol biosynthesis interaction might be used to develop new diagnostic and therapeutic targets in breast cancer treatment.

## Supplementary information

**Additional file 1: Figure S1.** NSDHL siRNA efficiently decreased NSDHL expression in MCF-7, BT-20 and MDA-MB-231 cells. **A, B, C** Data of relative expression levels of NSDHL mRNA in MCF-7, BT-20 and MDA-MB-231 cells transfected with NSDHL siRNA or control siRNA (10 nM and 20 nM); **D, E, F** Representative western blot image for NSDHL in MCF-7, BT-20 and MDA-MB-231 cells transfected with NSDHL siRNA or control siRNA (10 nM and 20 nM). Data represent the means ± standard deviations of three independent experiments, each performed in triplicates **p < 0.01, ***p < 0.001.

**Additional file 2: Figure S2.** Effect of NSDHL knockdown on viability, proliferation, and colony and sphere formation abilities of MCF-7 cells. **A, B** Data of cell viability and cell cycle in MCF-7 cells transfected with NSDHL siRNA or control siRNA (20 nM); **C, D** Representative images and data analyzed in colony formation of MCF-7 cells transfected with NSDHL siRNA or control siRNA (20 nM); **E** Representative image and data analyzed in 3D sphere formation of MCF-7 cells transfected with NSDHL siRNA or control siRNA (20 nM). *p < 0.05, **p < 0.01, ***p < 0.001.

**Additional file 3: Figure S3.** Effect of NSDHL knockdown on migratory abilities of MCF-7 cells. **A,B** Representative images and data analyzed in transwell migration assay and wound healing assay of MCF-7 cells transfected with NSDHL siRNA or control siRNA (20 nM). Data represent the means ± standard deviations of three independent experiments. *p < 0.05, **p < 0.01.

**Additional file 4: Figure S4.** NSDHL knockdown has no additional effect on erlotinib-induced cell death in MCF10A and MCF-7 cells. **A,B** Dose-response curve of erlotinib in MCF10A and MCF-7 cells transfected with NSDHL siRNA or control siRNA (20 nM). All data were expressed as means ± standard deviations.

## Data Availability

The datasets supporting the background of this article are included within additional files.

## Abbreviations

NSDHL: NAD(P)-dependent steroid dehydrogenase-like
siRNA: Short interfering RNA
EGFR: Epidermal growth factor receptor
shRNA: Short hairpin RNA
SREBP-1: Sterol regulatory element-binding transcription factor 1
qRT-PCR: Quantitative reverse transcription-polymerase chain reaction
IHC: Immunohistochemistry
GEO: Gene expression omnibus
RFS: Recurrence-free survival
HR: Hazard ratio
CI: Confidence interval
IC50: Half inhibitory concentration
SC4MOL: Sterol C4-methyl oxidase-like
FDFT1: Farnesyl-diphosphate farnesyltransferase 1
HMGCS1: 3-Hydroxy-3-methylglutaryl-CoA synthase 1
HMGCR: 3-Hydroxy-3-methylglutaryl-CoA reductase
EBP: Emopamil-binding protein
DHCR7: 7-Dehydrocholesterol reductase
LSS: Lanosterol synthase
HMG-CoA: 3-Hydroxy-3-methylglutaryl coenzyme-A
CYB5R3: Cytochrome B5 reductase 3
FDPS: Farnesyl diphosphate synthase
