# Supplementary figures and images for: NAD(P)-dependent steroid dehydrogenase-like is involved in breast cancer cell growth and metastasis
Source: BMC Cancer. 2020 May 4;20:375. doi: 10.1186/s12885-020-06840-2 (PMC7197182; doi:10.1186/s12885-020-06840-2)

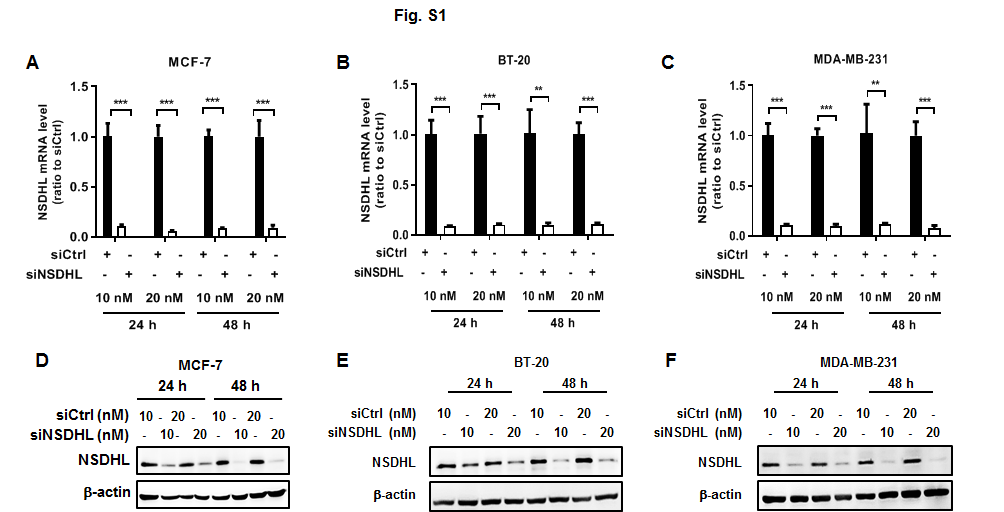

Supplement: Supplementary file 1 — Additional file 1: Figure S1. NSDHL siRNA efficiently decreased NSDHL expression in MCF-7, BT-20 and MDA-MB-231 cells. A, B, C Data of relative expression levels of NSDHL mRNA in MCF-7, BT-20 and MDA-MB-231 cells transfected with NSDHL siRNA or control siRNA (10 nM and 20 nM); D, E, F Representative western blot image for NSDHL in MCF-7, BT-20 and MDA-MB-231 cells transfected with NSDHL siRNA or control siRNA (10 nM and 20 nM). Data represent the means ± standard deviations of three independent experiments, each performed in triplicates **p < 0.01, ***p < 0.001. [file 12885_2020_6840_MOESM1_ESM.tif]

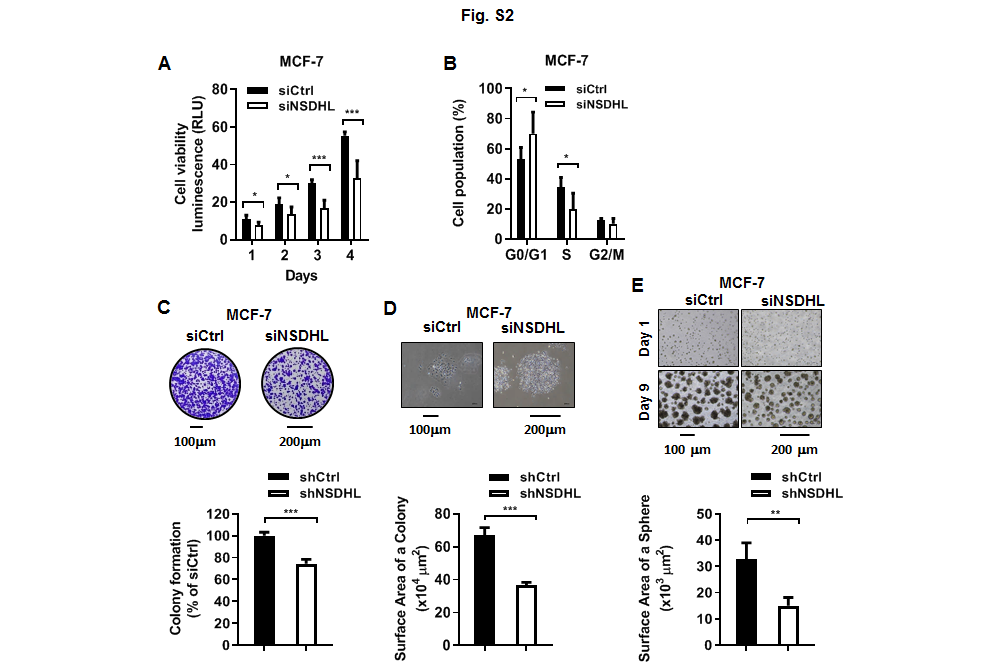

Supplement: Supplementary file 2 — Additional file 2: Figure S2. Effect of NSDHL knockdown on viability, proliferation, and colony and sphere formation abilities of MCF-7 cells. A, B Data of cell viability and cell cycle in MCF-7 cells transfected with NSDHL siRNA or control siRNA (20 nM); C, D Representative images and data analyzed in colony formation of MCF-7 cells transfected with NSDHL siRNA or control siRNA (20 nM); E Representative image and data analyzed in 3D sphere formation of MCF-7 cells transfected with NSDHL siRNA or control siRNA (20 nM). *p < 0.05, **p < 0.01, ***p < 0.001. [file 12885_2020_6840_MOESM2_ESM.tif]

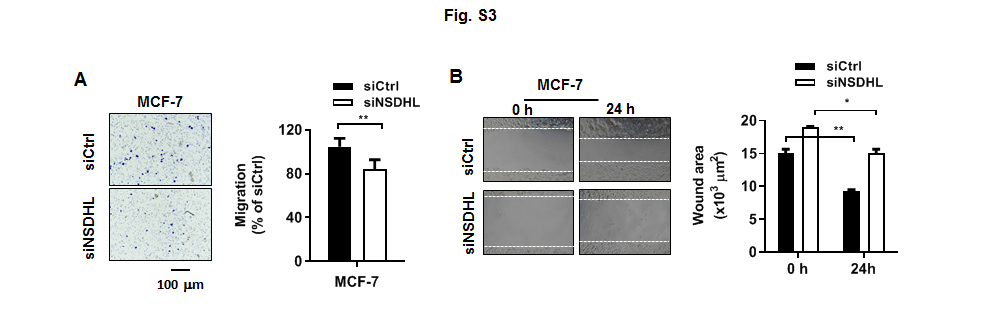

Supplement: Supplementary file 3 — Additional file 3: Figure S3. Effect of NSDHL knockdown on migratory abilities of MCF-7 cells. A,B Representative images and data analyzed in transwell migration assay and wound healing assay of MCF-7 cells transfected with NSDHL siRNA or control siRNA (20 nM). Data represent the means ± standard deviations of three independent experiments. *p < 0.05, **p < 0.01. [file 12885_2020_6840_MOESM3_ESM.tif]

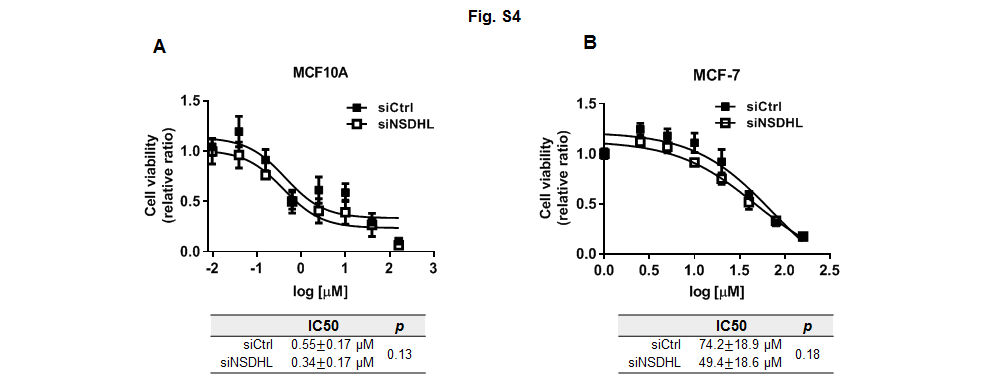

Supplement: Supplementary file 4 — Additional file 4: Figure S4. NSDHL knockdown has no additional effect on erlotinib-induced cell death in MCF10A and MCF-7 cells. A,B Dose-response curve of erlotinib in MCF10A and MCF-7 cells transfected with NSDHL siRNA or control siRNA (20 nM). All data were expressed as means ± standard deviations. [file 12885_2020_6840_MOESM4_ESM.tif]
